# Thymosin β4-Enhancing Therapeutic Efficacy of Human Adipose-Derived Stem Cells in Mouse Ischemic Hindlimb Model

**DOI:** 10.3390/ijms21062166

**Published:** 2020-03-21

**Authors:** Jong-Ho Kim, I-Rang Lim, Chi-Yeon Park, Hyung Joon Joo, Ji-Min Noh, Seung-Cheol Choi, Soon Jun Hong, Do-Sun Lim

**Affiliations:** Department of Cardiology, Cardiovascular Center, Korea University College of Medicine, Seoul 02841, Korea; mecey@naver.com (J.-H.K.); irang.lim@gmail.com (I.-R.L.); chiyeon19@gmail.com (C.-Y.P.); drjoohj@gmail.com (H.J.J.); wlals5344@gmail.com (J.-M.N.); choisc86@gmail.com (S.-C.C.); psyche94@gmail.com (S.J.H.)

**Keywords:** Thymosin β4, Adipose-derived stem cells, Endothelial Differentiation, Vascularization, Hindlimb ischemia

## Abstract

Thymosin β4 (Tβ4) is a G-actin sequestering protein that contributes to diverse cellular activities, such as migration and angiogenesis. In this study, the beneficial effects of combined cell therapy with Tβ4 and human adipose-derived stem cells (hASCs) in a mouse ischemic hindlimb model were investigated. We observed that exogenous treatment with Tβ4 enhanced endogenous *TMSB4X* mRNA expression and promoted morphological changes (increased cell length) in hASCs. Interestingly, Tβ4 induced the active state of hASCs by up-regulating intracellular signaling pathways including the PI3K/AKT/mTOR and MAPK/ERK pathways. Treatment with Tβ4 significantly increased cell migration and sprouting from microbeads. Moreover, additional treatment with Tβ4 promoted the endothelial differentiation potential of hASCs by up-regulating various angiogenic genes. To evaluate the in vivo effects of the Tβ4-hASCs combination on vessel recruitment, dorsal window chambers were transplanted, and the co-treated mice were found to have a significantly increased number of microvessel branches. Transplantation of hASCs in combination with Tβ4 was found to improve blood flow and attenuate limb or foot loss post-ischemia compared to transplantation with hASCs alone. Taken together, the therapeutic application of hASCs combined with Tβ4 could be effective in enhancing endothelial differentiation and vascularization for treating hindlimb ischemia.

## 1. Introduction

Adipose-derived stem cells (ASCs) have several advantages due to easy accessibility via minimally invasive liposuction of the stromal vascular fraction of adipose tissues and easy in vitro expansion. ASCs are capable of differentiating into diverse types of mesenchymal cells including endothelial lineages [1,2]. Increasing evidence demonstrated that ASCs have the potential to augment neovascularization and improve functional recovery post-ischemia [3,4].

Thymosin β4 (Tβ4) is a water-soluble and highly conserved protein comprising of 43 amino acids (MW: 4964 Da) [5,6]. It is reported to regulate the actin cytoskeleton by sequestering G-actin [7]. Tβ4 is a ubiquitous protein expressed in many cells and tissues and has been shown to mediate numerous biological processes including blood vessel formation and wound healing [8]. Previous studies reported that Tβ4 increases the proliferation, migration, and adhesion of endothelial progenitor cells and also inhibits apoptosis and cellular senescence in vitro [9,10]. Moreover, Tβ4 was reported to promote the angiogenesis potential of endothelial cells and infarcted hearts by up-regulating vascular endothelial growth factor (VEGF) expression [11,12]. Overall, previous findings suggest that Tβ4 may have significant potential for influencing the endothelial differentiation of mesenchymal stem cells (MSCs). Several studies demonstrated MSC differentiation into an endothelial lineage using VEGF or fibroblast growth factor 2 (FGF-2); [13,14,15] however, endothelial differentiation using Tβ4 has not been reported yet.

N-terminal sequence, one of the three main functional domains of Tβ4, is composed of a tetrapeptide (ac-SDKP) that is known to have anti-inflammatory and anti-fibrotic properties. These properties of Tβ4 have been reported to promote cell survival and inhibit apoptosis [16,17]. In addition, Tβ4 has been shown to facilitate cardioprotection by attenuating cardiac fibrosis post-myocardial infarction (post-MI) and regeneration of the ischemic heart by promoting cardiomyocyte survival [18,19,20]. It has also been reported that Tβ4 modulates the inflammatory environment within the infarcted region of the heart by reducing leukocyte infiltration after MI [21,22].

The purpose of the present study was to investigate whether the endothelial differentiation potential and therapeutic efficacy of ASC transplantation could be further enhanced by Tβ4 treatment. In the present study, exogenous treatment with Tβ4 was found to not only induce morphological changes, but also enhance the angiogenic sprouting of ASCs. Additionally, we revealed that the combination of ASCs and Tβ4 further promoted microvessel recruitment and enhanced blood flow post-hindlimb ischemia.

## 2. Results

### 2.1. Treatment with Tβ4 Promotes Morphological Changes in hASCs

**Figure 1:**
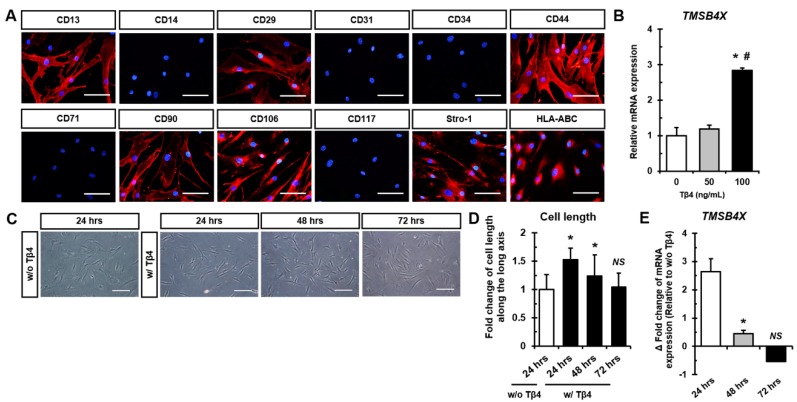
Treatment with Tβ4 promotes morphological changes in hASCs. (**A**) Immunofluorescence images of cell surface markers in hASCs indicate positive expression for CD13, CD29, CD44, CD90, CD106, Stro-1, and HLA-ABC and negative expression for CD14, CD31, CD34, CD71, and CD117. Scale bar = 100 μm. (**B**) Real-time PCR analysis of relative endogenous *TMSB4X* mRNA expression in hASCs after 24 h of dose-dependent exogenous treatment with Tβ4 (0, 50, and 100 ng/mL). * *p* < 0.05 vs. 0 ng/mL; ^#^ *p* < 0.05 vs. 50 ng/mL. (**C**) Representative phase-contrast images of hASCs at 24 h, 48 h, and 72 h after a single Tβ4 treatment (100 ng/mL). Scale bar = 100 μm. (**D**) Elongated cell length after a single treatment with Tβ4 at 24 h and 48 h compared to cell length of untreated cells. * *p* < 0.05 vs. without Tβ4 treatment. (**E**) Changes in *TMSB4X* mRNA expression upon a single Tβ4 treatment. Data are shown as Δ (fold change of 2^−∆∆Ct^ values at 24 h, 48 h, and 72 h after Tβ4 treatment compared to that without Tβ4 treatment). * *p* < 0.05 vs. 2^−∆∆Ct^ values of without Tβ4 treatment; *NS*, not significant; w/ Tβ4, with Tβ4 treatment; w/o Tβ4, without Tβ4 treatment.

In this study, human ASCs (hASCs) were found to be positive for CD13, CD29, CD44, CD90, CD106, Stro-1, and HLA-ABC expression (Figure 1A). Furthermore, hASCs were treated with recombinant Tβ4 protein and 100 ng/mL of Tβ4 was observed to significantly up-regulate endogenous *TMSB4X* mRNA expression compared to the expression in untreated cells (Figure 1B). Interestingly, in Figure 1C, treatment with Tβ4 was observed to increase the cell length of hASCs after 24 h compared to the cell length of hASCs without Tβ4 treatment (w/o Tβ4). A single treatment with Tβ4 significantly increased cell length after 24 and 48 h compared to that observed without Tβ4. However, we observed that cell length of Tβ4-treated hASCs at after 72 h was similar to that of hASCs without Tβ4 treatment (Figure 1D). Consistently, *TMSB4X* mRNA levels after 24 and 48 h of a single treatment with Tβ4 were found to be significantly elevated compared to those in hASCs without Tβ4 treatment (Figure 1E). Conversely, *TMSB4X* siRNA significantly reduced *TMSB4X* mRNA expression; however, it did not affect the cell length of hASCs (Figure S1A–C). Thus, considering the upholding of the endogenous Tβ4 level and the morphological effect of Tβ4 treatment, we established 24 h as a suitable Tβ4 treatment time for further experiments (within 48 h) to investigate the cellular effects of Tβ4 in hASCs.

### 2.2. Tβ4 Induces the Active State of hASCs

**Figure 2:**
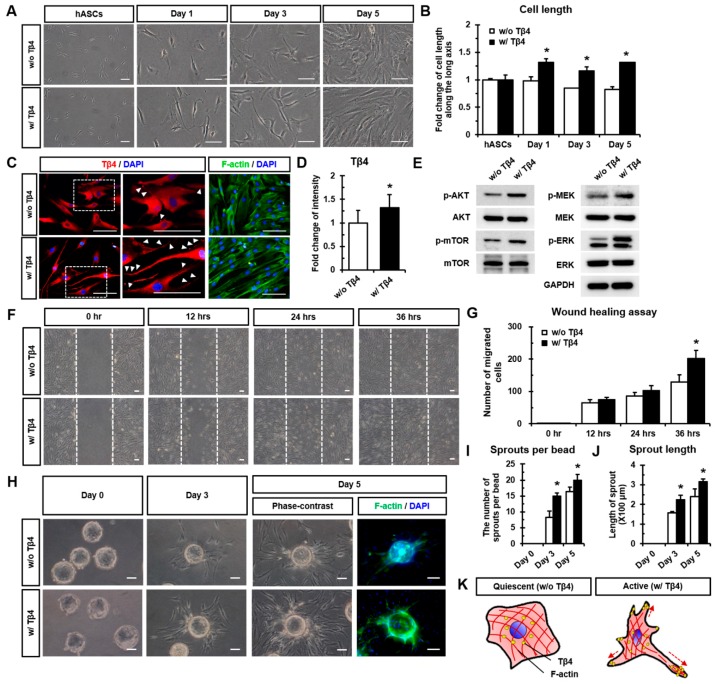
Tβ4 induces the active state of hASCs. (**A**) Representative phase-contrast images and (**B**) Quantitative analysis of cell length along the long axis of hASCs after long-term exogenous treatment with Tβ4 (100 ng/mL). Scale bar = 100 μm. * *p* < 0.05 vs. without Tβ4 at each time point. (**C**) Immunofluorescence images and (**D**) Quantitative analysis of fluorescence intensities of Tβ4 after 24 h of treating hASCs in combination with Tβ4. Actin cytoskeleton (F-actin) was stained with phalloidin. White arrowheads indicate the elongation of the cell body upon Tβ4 expression. Scale bar = 100 μm. * *p* < 0.05 vs. without Tβ4. (**E**) Activation of the AKT/mTOR and MEK/ERK signaling pathways in hASCs upon Tβ4 treatment confirmed by western blot. Quantitative analysis of band intensities is illustrated in Figure S2A. (**F**) Representative phase-contrast images of wound healing assay at different time points. Cells were wounded and treated with Tβ4 at 0 h. Scale bar = 100 μm. (**G**) Quantification of the migrated cell number at 0, 12, 24 h, and 36 h after scratching. * *p* < 0.05 vs. without Tβ4 treatment at each time point. (**H**) Microbead sprouting assay of hASC-coated microbeads. Beads were seeded on Matrigel and treated with Tβ4. At day 5, hASC-coated microbeads were stained for F-actin. Scale bar = 100 μm. (**I**) Number of sprouts per bead and (**J**) sprout length were measured using phase-contrast images. * *p* < 0.05 vs. without Tβ4 at each time point. (**K**) Schematic diagram depicts the active state of hASCs upon exogenous treatment with Tβ4 via morphological alterations and activation of intracellular signaling pathways. w/ Tβ4, with Tβ4 treatment; w/o Tβ4, without Tβ4 treatment.

We investigated the upholding of Tβ4-induced elongated cell length every 48 h (at day 0, day 2, and day 4). Long-term treatment with Tβ4 was found to sustain the significantly increased cell length of hASCs at day 1, day 3, and day 5 (at 24 h after treatment) compared to without Tβ4 treatment (Figure 2A,B). After 24 h of treatment with Tβ4, hASCs showed an elongated cell body as shown in actin cytoskeleton (F-actin)-stained images and expression of Tβ4 (red) in cell apexes (indicated by white arrowheads in magnified images in Figure 2C). In addition, the fluorescence intensity of Tβ4 expression in hASCs was significantly higher upon Tβ4 treatment than that without Tβ4 treatment (Figure 2D). Interestingly, treatment with Tβ4 significantly enhanced the phosphorylation of AKT, mTOR, MEK, and ERK compared to that without Tβ4 treatment (Figure 2E and Figure S2). Moreover, the wound healing assay revealed that treating hASCs with Tβ4 promoted cell migration in a time-dependent manner (Figure 2F,G). Furthermore, we evaluated the effects of Tβ4 on cell sprouting using microbeads covered with hASCs (Figure 2H). Compared to treatment without Tβ4, the sprout number and length were significantly increased on both day 3 and day 5 after treatment with Tβ4 (Figure 2I,J). Collectively, Tβ4 induced the active state of hASCs by up-regulating intracellular signaling pathways and increasing the migration and sprouting of cells, which showed an elongated morphology as depicted in Figure 2K.

### 2.3. Additional Treatment with Tβ4 Enhances Endothelial Differentiation of hASCs

**Figure 3:**
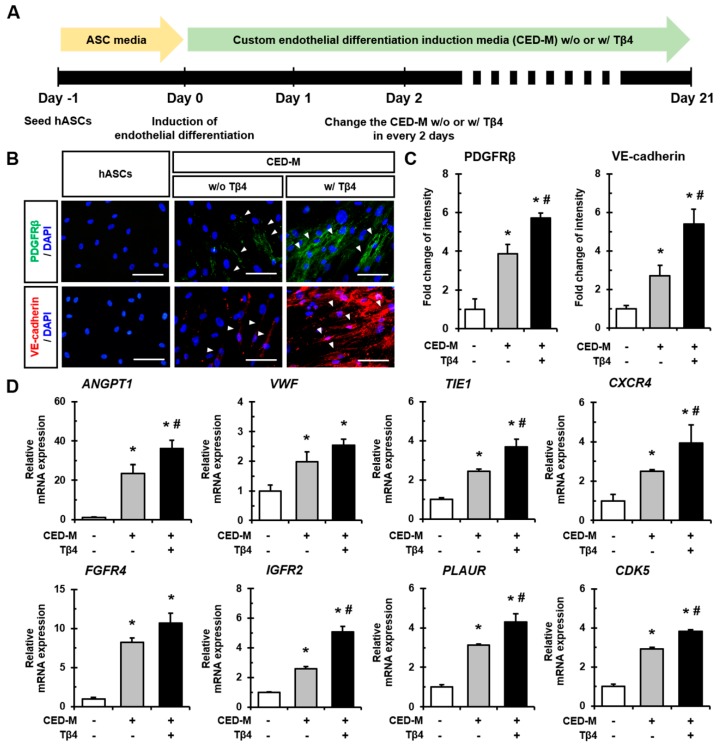
Additional treatment with Tβ4 enhances endothelial differentiation of hASCs. (**A**) Schematic diagram showing the induction of endothelial differentiation of hASCs using CED-M and additional Tβ4 treatment for 21 days. (**B**) Representative immunofluorescence images of PDGFRβ and VE-cadherin after inducing endothelial differentiation without or with Tβ4 treatment. White arrowheads indicate positive expression of PDGFRβ and VE-cadherin. Scale bar = 100 μm. (**C**) Quantitative analysis of fluorescence intensities of PDGFRβ and VE-cadherin. * *p* < 0.05 vs. CED-M^-^/Tβ4^-^; ^#^ *p* < 0.05 vs. CED-M^+^/Tβ4^+^; -, non-treated; +, treated. (**D**) Real-time PCR analysis of expression of various genes including *ANGPT1*, V*WF*, *TIE1*, *CXCR4*, *FGFR4*, *IGFR2*, *PLAUR*, and *CDK5* after 21 days of endothelial differentiation induction. * *p* < 0.05 vs. CED-M^-^/Tβ4^-^; ^#^ *p* < 0.05 vs. CED-M^+^/Tβ4^+^; -, non-treated; +, treated; w/ Tβ4, with Tβ4 treatment; w/o Tβ4, without Tβ4 treatment.

We next investigated whether Tβ4 could influence the endothelial differentiation of hASCs. Endothelial differentiation was induced using custom endothelial differentiation induction media (CED-M), and the medium was additionally treated with Tβ4 every 48 h (Figure 3A). After 21 days of induction, hASCs cultured in CED-M were found to have an increased expression of PDGFβ and VE-cadherin compared to hASCs. In addition, the expression of these proteins was significantly promoted upon additional treatment with Tβ4 (Figure 3B,C). Indeed, the mRNA expression of various angiogenic factors and receptors including *ANGPT1*, *VWF*, *TIE1*, *CXCR4*, *FGFR4*, *IGFR2*, *PLAUR*, and *CDK5* was found to be significantly increased upon CED-M-mediated differentiation induced by Tβ4 treatment compared to that observed in normal hASCs and cells that underwent CED-M-mediated differentiation without Tβ4 treatment (Figure 3D). Therefore, additional treatment with Tβ4 promoted endothelial differentiation of hASCs with elevated expression of angiogenesis-related genes.

### 2.4. Transplantation of hASCs in Combination with Tβ4 Improves Blood Vessel Recruitment and Blood Flow After Ischemia

**Figure 4:**
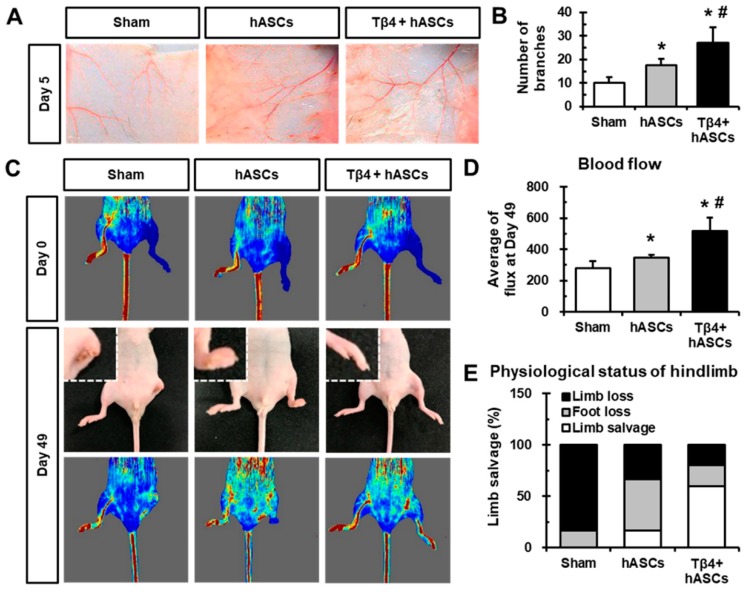
Transplantation of hASCs in combination with Tβ4 improves blood vessel recruitment and blood flow after ischemia. (**A**) Representative images of mouse dorsal window chamber model after 5 days of transplantation of chambers containing hASCs without or with Tβ4. (**B**) Quantification of the number of microvessel branches per chamber depending upon the transplantation condition. * *p* < 0.05 vs. Sham; ^#^ *p* < 0.05 vs. hASCs. *n* = 3 rats in each group. (**C**) Representative Laser Doppler images after induction of hindlimb ischemia (day 0) and after 49 days of transplantation. Magnified inlet images show limb loss in the sham group and foot loss in the hASCs group. Sham (*n* = 6); hASCs (*n* = 5); Tβ4 + hASCs (*n* = 6). (**D**) Measurement of blood flow at day 49, as measured by Laser Doppler images. * *p* < 0.05 vs. Sham; ^#^ *p* < 0.05 vs. hASCs. (**E**) Percentage of physiological status of hindlimb after 49 days of ischemia and transplantation. * *p* < 0.05 vs. Sham; ^#^ *p* < 0.05 vs. hASCs.

To evaluate the in vivo angiogenic effect of combinatorial treatment with hASCs and Tβ4 on recruiting vessels, window chambers containing hASCs without or with Tβ4 were transplanted onto the dorsal skin of mice. At day 5 post-transplantation, the transplanted sites were opened and microvessel formation was evaluated (Figure 4A). We found that mice transplanted with hASCs and Tβ4 showed a significantly increased number of microvessel branches compared to mice treated with only cell culture media (sham group) and hASCs (Figure 4B). Moreover, hindlimb ischemia was induced by ligating the left femoral arteries of nude mice, and hASCs without or with Tβ4 were injected. Furthermore, blood flow was assessed using Laser Doppler after day 0, day 7, day 21 (data not shown), and day 49 of the procedure (Figure 4C). Transplantation of hASCs in combination with Tβ4 caused significant blood flow in the hindlimb compared to that caused by transplantation of only cell culture media (sham group) and hASCs without Tβ4, as indicated by average flux values (Figure 4D). In addition, treatment with hASCs in combination with Tβ4 attenuated limb or foot loss compared to the sham group (Figure 4E). Therefore, the higher blood vessel recruitment and improvement of ischemia were observed when mice were injected with a combination of hASCs and Tβ4.

## 3. Discussion

The novel findings of our study were as follows: (i) the effects on cell morphology and underlying mechanisms by exogenous treatment with Tβ4 to hASCs, (ii) the role of Tβ4 in endothelial differentiation, and (iii) enhanced therapeutic efficacy of hASCs combined with Tβ4 for treating mouse hindlimb ischemia.

Actin sequestration and desequestration have been reported to be dynamically controlled by various actin-binding proteins such as Tβ4 [23]. We found that *TMSB4X* knockdown using siRNA significantly decreased the expression of other actin polymerization-associated proteins such as Rac1/Cdc42, WAVE-2, N-WASP, and ARP3 compared to their expression in normal hASCs (Figure S1D,E). This finding reemphasizes the significant contribution of Tβ4 in actin dynamics. In addition, actin cytoskeletal rearrangement is required for morphology, motility, and migration of adult stem cells in response to their microenvironment [24]. Indeed, our results revealed that Tβ4 treatment induced cell elongation of hASCs to begin to sprout and migrate (Figure 2A–C,K). Such alterations resulted in significant increases of cell migration and sprouting of hASCs from microbeads upon Tβ4 treatment (Figure 2F–I). On the contrary, Xiao et al. [17] reported that the depletion of Tβ4 promotes migration, proliferation, and activation of hepatic stellate cells. We confirm that *TMSB4X* knockdown using siRNA significantly decreased the proliferation rate compared to normal hASCs (Figure S1F). However, in other cell types, such as palatal cells [25], hair follicle stem cells [26], and cardiac cells [20], Tβ4 has been shown to stimulate cell migration and wound healing. Overexpression of Tβ4 has been proved to enhance cell proliferation, invasion, and expression of epithelial-to-mesenchymal transition (EMT)-inducing transcription factor, Twist-related protein 1 (Twist1) in cancer cells [27].

Furthermore, we investigated the intracellular mechanisms underlying the proliferation and survival of hASCs upon exogenous Tβ4 treatment. As shown in Figure 2D,E, Tβ4 treatment was found to significantly increase the phosphorylation of AKT/mTOR and MEK/ERK in hASCs. These results are supported by previous studies that report augmented cell migration and survival of Tβ4-overexpressing cancer cells via the Tβ4-mediated up-regulation of AKT and interleukin-linked kinase (ILK) [28,29]. Consistently, apoptosis activated by Fas ligand and hydrogen peroxide has been shown to be markedly suppressed by Tβ4 in human corneal epithelial cells, indicating the anti-apoptotic effect of Tβ4 [7]. Additionally, it has been reported that Tβ4 enhances MSC proliferation [30] and interleukin-8 (IL-8) secretion by activating the ERK and NF-κB pathways [31].

A previous study showed that Tβ4 enhances adipogenic differentiation and inhibits osteogenic differentiation of bone marrow-derived MSCs by regulating F-actin formation [32]. During the capillary-like tube formation of endothelial cells, endogenous Tβ4 levels increase [33], and exogenous Tβ4 treatment has been shown to facilitate tube formation and sprouting from coronary artery rings [12]. Here, we demonstrated that the addition of Tβ4 markedly increased the endothelial differentiation of hASCs (Figure 3). Among Tβ4-mediated up-regulated angiogenic genes (Figure 3D), specifically, the activation of *Ang-1* signaling plays an important role in enhancing the therapeutic effect of Tβ4. Wang et al. [34] reported that inactivating Ang-1 using a neutralizing antibody against Tie2 inhibited the Tβ4-induced improvement of neurovascular function and remodeling in mice with diabetic peripheral neuropathy.

Tβ4 has been reported to be involved in vascular development [35] and healing liver fibrosis [5,36,37]. Specifically, during cardiovascular development, the cardiac-specific knockdown of *TMSB4X* has been shown to cause various defects in heart development and has also been proved to be essential for embryonic coronary vasculogenesis [38,39]. Moreover, Tβ4 facilitates angiogenesis and neovascularization in ischemic hearts resulting in enhanced functional recovery post-MI [40]. In this study, we used ASCs, which have been shown to have therapeutic potential for treating hindlimb ischemia [41,42]. Transplantation of hASCs in combination with Tβ4 treatment was found to significantly alleviate limb salvage and blood flow after 49 days of ischemia compared to transplantation of hASCs alone (Figure 4C–E) and treatment with Tβ4 alone (data not shown). Thus, our results suggest that combined treatment with Tβ4 enhances the therapeutic revascularization potential of hASCs. Additionally, further experiment will be required as a mechanism study of blood vessel recruitment and improvement of blood flow by transplanted-Tβ4 at host tissue, and synergistic effect of Tβ4 with transplanted cells.

Through the present study, we defined the mechanisms underlying Tβ4-mediated changes in morphological features, increase in cell migration, and sprouting of ASCs. Furthermore, we suggested that Tβ4 might be an important factor for differentiating ASCs into endothelial lineages by augmenting angiogenesis-related gene expression. Moreover, the combination of Tβ4 treatment with hASC transplantation can be developed as a more effective alternative therapeutic approach for treating peripheral arterial diseases through ASC transplantation.

## 4. Materials and Methods

### 4.1. Cell Source

Human ASCs (hASCs) were purchased (PT-5006, Lonza, Walkersville, MD, USA) and cultured using the human MesenCult proliferation kit (#05411, STEMCELL Technologies, Vancouver, BC, Canada) containing 100 U/mL penicillin/streptomycin (P/S; 15140, Gibco, Waltham, MA, USA). Cells were incubated in a humidified chamber with 5% CO_2_ at 37 °C. hASCs were treated with recombinant human Tβ4 (#140-14, Peprotech, Rocky Hill, NJ, USA) in Dulbecco’s modified Eagle’s medium (DMEM) with low glucose (SH30021, Hyclone) containing 2% fetal bovine serum (FBS; #16000-044, Invitrogen, Waltham, MA, USA) and P/S. Phase-contrast images were obtained using an upright fluorescence microscope (DMI 300B, Leica Microsystems, Wetzlar, Germany). Cell length along the long axis was evaluated using the ImageJ software (v1.32, National Institute of Health, Bethesda, MO, USA).

### 4.2. Real-Time Polymerase Chain Reaction

Total RNA was extracted from each sample using TRI reagent (TR118, Molecular Research Center, Cincinnati, OH, USA), and cDNA was synthesized as described previously [43]. Real-time polymerase chain reaction (real-time PCR) was performed using the iQ^TM^ SYBR Green Supermix and My iQ^TM^ 2 real-time PCR detection system (both from Bio-Rad, Hercules, CA, USA). The anti-human PCR primer sequences are listed in Table S1. Relative gene expression levels were quantified using the 2^−∆∆Ct^ method and normalized to *GAPDH* expression.

### 4.3. Immunofluorescence Staining

First, hASCs were fixed with 2% paraformaldehyde (PFA; P6148, Sigma-Aldrich, St. Louis, MO, USA) and blocked with 5% normal goat serum (NGS; 16210, Gibco) in phosphate-buffered saline + 0.1% Tween 20 (PBST). Next, cells were incubated for 1 h at room temperature (RT) with the following primary antibodies: CD13 (#555393), CD14 (#555396), CD29 (#555442), CD31 (#555444), CD34 (#550760), CD44 (#550988), CD71 (#555534), CD90 (#555593), CD106 (#555645), CD117 (#555713), and vascular endothelial-cadherin (VE-cadherin; #555661) obtained from BD Biosciences (San Jose, CA, USA); Stro-1 (ab57834), human leukocyte antigen -A, -B, -C (HLA-ABC; ab70328), and platelet-derived growth factor receptor β (PDGFRβ; ab32570) obtained from Abcam (Cambridge, UK); and Tβ4 (AB6019) obtained from Merck Millipore. After washing with PBST, cells were incubated for 1 h at RT with the corresponding secondary antibodies: anti-mouse IgG Alexa Fluor 594 conjugate (A11005), anti-rabbit IgG Alexa Fluor 594 conjugate (A11012), and anti-rabbit IgG Alexa Fluor 488 conjugate (A21441) obtained from Invitrogen. Actin cytoskeleton (F-actin) was stained with Alexa Fluor 488 phalloidin (A12379, Invitrogen). Nuclei were counterstained with 4’,6-diamidino-2-phenylindole (DAPI; D9542, Sigma-Aldrich). Immunofluorescence images were captured using the TE-FM Epi-fluorescence system attached to an inverted microscope (BX61, Olympus, Tokyo, Japan). The fluorescence intensities of Tβ4, PDGFRβ, and VE-cadherin were quantified using the ImageJ software.

### 4.4. Western Blot Analysis

Proteins were extracted from hASCs treated without or with Tβ4 for 24 h using cell lysis buffer (#9803, Cell Signaling Technologies, Danvers, MA, USA) containing 1 mM phenylmethylsulfonyl fluoride (PMSF). Equal amounts of protein were resolved by 10% sodium dodecyl sulfate-polyacrylamide gel electrophoresis (SDS-PAGE) and transferred onto polyvinylidene difluoride membranes (PVDF; 10600023, GE Healthcare, Chicago, IL, USA). Membranes were blocked for 1 h in 5% (*w/v*) skim milk in tris-buffered saline (TBS; T2008, Biosesang, Gyeonggi, Korea) + 0.1% Tween-20 and incubated overnight at 4 °C with the following primary antibodies: phospho-protein kinase B (p-AKT; #9271), phospho-mammalian target of rapamycin (p-mTOR; #2971), mTOR (#2972), phospho-mitogen activated protein kinase (p-MEK; #9121), MEK (#9122), phospho-extracellular receptor kinase (p-ERK; #9106), ERK (#9102), ras-related C3 botulinum toxin substrate 1 (Rac1)/cell division cycle 42 (Cdc42) (#4651), WASP family protein member 2 (WAVE-2) (#3659), neural WASP (N-WASP) (#4848), actin-related protein 2 (ARP2) (#3128), ARP3 (#4738) obtained from Cell Signaling Technologies; AKT (sc-1618) obtained from Santa Cruz (Dallas, TX, USA); and GAPDH (G8795) obtained from Sigma-Aldrich. Following washing, membranes were incubated with anti-rabbit (#7074), anti-mouse (#7076, both from Cell Signaling Technologies), or anti-goat (HAF109, R&D Systems, Minneapolis, MN, USA) HRP-conjugated secondary antibodies for 1 h at RT. Protein bands were detected using the Clarity Western ECL Substrate (#1705061) and ChemiDoc Touch Imaging System (#1708370, both from Bio-Rad). Band intensities were analyzed using Quantity One software (Bio-Rad, Hercules, CA, USA).

### 4.5. Wound Healing Assay

After attachment, a wound was created across the center of the dish, detached cells were removed by washing twice, and attached cells were treated with 100 ng/mL of Tβ4. Cell migration was observed at 0 h, 12 h, 24 h, and 36 h after scratching and treatment. Number of cells that migrated across the scratch line were counted using phase-contrast images.

### 4.6. Microbead Sprouting Assay

Autoclaved Cytodex-3 (C3275, Sigma-Aldrich) was coated with 50 × 10^4^ hASCs per 2500 microbeads. After 3 h of incubation with gentle mixing every 20 min, hASC-coated microbeads were seeded on Matrigel and treated with Tβ4 in 5% DMEM. Phase-contrast images were captured at day 0, day 3, and day 5 to observe the sprouting from the microbeads. The number and length of sprouts were quantified using the ImageJ software. At day 5, microbeads were fixed and stained with actin cytoskeleton (F-actin) and DAPI to visualize cell sprouts.

### 4.7. Induction of Endothelial Differentiation

hASCs were plated at a cell density of 5 × 10^4^ in 12-well plates and allowed to attach. Custom endothelial differentiation induction media (CED-M) was composed of ascorbic acid (A8960), sodium pyruvate (#11360070), L-glutamine (G7513, all from Sigma-Aldrich), 50 ng/mL of FGF-2 (234-FSE, R&D Systems), 10% FBS, and P/S in RPMI-1640 (12-702F, Lonza). CED-M was replaced every 2 days without or with Tβ4. After 21 days of endothelial differentiation induction, cells were analyzed by immunofluorescence staining and real-time PCR.

### 4.8. Dorsal Window Chamber Assay

All procedures were approved by the Institutional Animal Care and Use Committee of Korea University College of Medicine for animal research (27 Jun 2016; No. KOREA-2016-0124). Male C57BL/6J mice were obtained from Orient Experimental Animal Laboratory (Gyeonggi, Korea).

Male C57BL/6J mice randomly assigned to the following three groups (*n* = 3 mice per group). Window chambers (PR0001401) were covered by membrane filters (pore size = 0.45 µM; HAWP01300, both from Millipore, Burlington, MA, USA) and filled with hASCs (5 × 10^4^) without or with 100 ng/mL of Tβ4 in 5% DMEM. All mice were anesthetized with a mixture of ketamine (44 mg/kg; Yuhan, Seoul, Korea) and xylazine hydrochloride (0.75 mg/kg; Rompun, Bayer, Leverkusen, Germany) and shaved. After skin incision, chambers were subcutaneously implanted into the dorsal side of mice. After 5 days of implantation, mice were anesthetized and images of dorsal sides of mice were taken. The number of subcutaneous microvessels per mouse was quantified.

### 4.9. Mouse Hindlimb Ischemia Model

Seventeen six-week-old male nude mice were obtained from Orient Experimental Animal Laboratory and anesthetized with a mixture of ketamine and xylazine hydrochloride. All mice were subjected to surgical ligation of left proximal femoral arteries. hASCs (1 × 10^6^) were transplanted without or with Tβ4 (5 mg/kg) via intramuscular injection at three different sites around the ligated arteries. The right leg was used as a control. After induction of ischemia (day 0) and 49 days after transplantation, blood flow was evaluated using the Laser Doppler imager and software (both from Moor Instruments, Devon, UK).

### 4.10. Statistical Analysis

All statistical values are expressed as mean ± standard deviation (SD). Analysis of variance (ANOVA) was used to compare normally distributed data of all groups. Significant differences between means were determined using ANOVA followed by Student-Newman-Keuls test. All P-values are two-sided, and * *p* < 0.05 was considered statistically significant. All statistical analyses were performed using the Sigma Stat software (Ver. 3.1, Systat Software, San Jose, CA, USA).

## Supplementary Materials

The following are available online at https://www.mdpi.com/1422-0067/21/6/2166/s1, Figure S1: Effects of TMSB4X knockdown in hASCs. (A) Relative mRNA expression of TMSB4X after 24 h of transfection with ncRNA or TMSB4X siRNA (100 nM), as evaluated by real-time PCR. **p* < 0.05 vs. hASCs; #*p* < 0.05 vs. ncRNA. (B) Representative phase-contrast images and (C) quantitative analysis of cell length along the long axis after 24 h of transfection with ncRNA or TMSB4X siRNA. Scale bar = 100 µm; NS, not significant. (D, E) Western blot analysis of proteins associated with actin polymerization, such as Ras-related C3 botulinum toxin substrate 1 (Rac1)/cell division cycle 42 (Cdc42), WASP family protein member 2 (WAVE-2), neural WASP (N-WASP), actin-related protein 2 (ARP2), and ARP3 in hASCs and TMSB4X siRNA-transfected hASCs. Band intensities were normalized to that of GAPDH. (F) Cell proliferation of hASCs and TMSB4X siRNA-transfected hASCs were evaluated by cell count at 3, 6, 9, 12, 24, and 36 h after seeding. **p* < 0.05 vs. hASCs, Figure S2: Quantification of western blot results. Band intensity of each phosphorylated protein was normalized to that of the respective total protein (Figure 2E). **p* < 0.05 vs. w/o Tβ4. w/ Tβ4, with Tβ4 treatment; w/o Tβ4, without Tβ4 treatment. Table S1: Real-time polymerase chain reaction primer information.
