# Supplementary material for: Thymosin β4-Enhancing Therapeutic Efficacy of Human Adipose-Derived Stem Cells in Mouse Ischemic Hindlimb Model
Source: Int J Mol Sci. 2020 Mar 21;21(6):2166. doi: 10.3390/ijms21062166 (PMC7139370; doi:10.3390/ijms21062166)
Supplement: Supplementary file 1 [file ijms-21-02166-s001.pdf]

Supplementary Materials

Thymosin β4-enhancing Therapeutic Efficacy of Human Adipose-derived Stem Cells in Mouse Ischemic Hindlimb Model

Short Title: Thymosin β4-enhancing Therapeutic Efficacy of ASCs

Jong-Ho Kim[1,†], I-Rang Lim[1,†], Chi-yeon Park[1], Hyung Joon Joo[1], Seung-Cheol Choi[1], Ji-Min Noh[1], Soon Jun Hong[1], Do-Sun Lim[1,*]

[1]Department of Cardiology, Cardiovascular Center, Korea University College of Medicine, Seoul, Republic of Korea.

[†]These authors contributed equally.

Contents

1. Supplementary Materials and Methods
2. Supplementary Table (Table S1)
3. Supplementary Figure and Figure Legend (Figure S1 and S2)

1. Supplementary Materials and Methods

1.1 Transfection of TMSB4X

To knock down *TMSB4X* mRNA expression, hASCs were transfected for 24 hrs with 100 nM of human *TMSB4X* small interfering RNA (siRNA; L-011408-01-005, Dharmacon) or negative control siRNA (ncRNA; SN-1003, Bioneer) using Lipofectamine RNAiMAX (#13778, Invitrogen) after cell attachment. Knockdown efficiency was confirmed by real-time PCR. Cell length along the long axis was evaluated using phase-contrast images and the ImageJ software. After 24 hrs of transfection, total proteins from non-transfected and transfected cells were extracted and analyzed by western blotting. Antibodies used for western blot analysis are listed in Material and Methods.

2. Supplementary Table (Table S1)

Table S1. Real-time polymerase chain reaction primer information

Protein	*Gene*	*Primer sequence*	
		**Forward primer (5′-3′)**	**Reverse primer (5′-3′)**
Tβ4	***TMSB4X***	ACGCAAGAGAAAAATCCACTG	GCACGCCTCATTACGATTC
Ang-1	***ANGPT1***	GAAGGGAACCGAGCCTATTC	AGGGCACATTTGCACATACA
vWF	***VWF***	TAAGTCTGAAGTAGAGGTGG	AGAGCAGCAGGAGCACTGGT
Tie1	***TIE1***	GCTAGAGTGGGAGGACATCACCTTTG	CTCCCGCAAAGTCACGATGGTCATT
CXCR4		CCTGCCTGGTATTGTCATCC	AGGATGACTGTGGTCTTGAGG
FGFR4		ATTCCATCGGCCTCTCCTAC	TAGCAAAGTGGGAGACTTGGT
IGFR2		CTGCAGCGAGAGCCAAGT	CGTTGCTCTTCATGGGACA
uPAR	***PLAUR***	GTAGCCACCGGCACTCAC	TGGTCTCAGGGCAGTAGTACTTT
VE-Cadherin	***CDK5***	GGCATCTTCGGGTTGATCCT	CCGACAGTTGTAGGCCCTGTT
GAPDH		GAGTCCACTGGCGTCTTCAC	TTCACACCCATGACGAACAT

3. Supplementary Figure and Figure Legend (Figure S1)

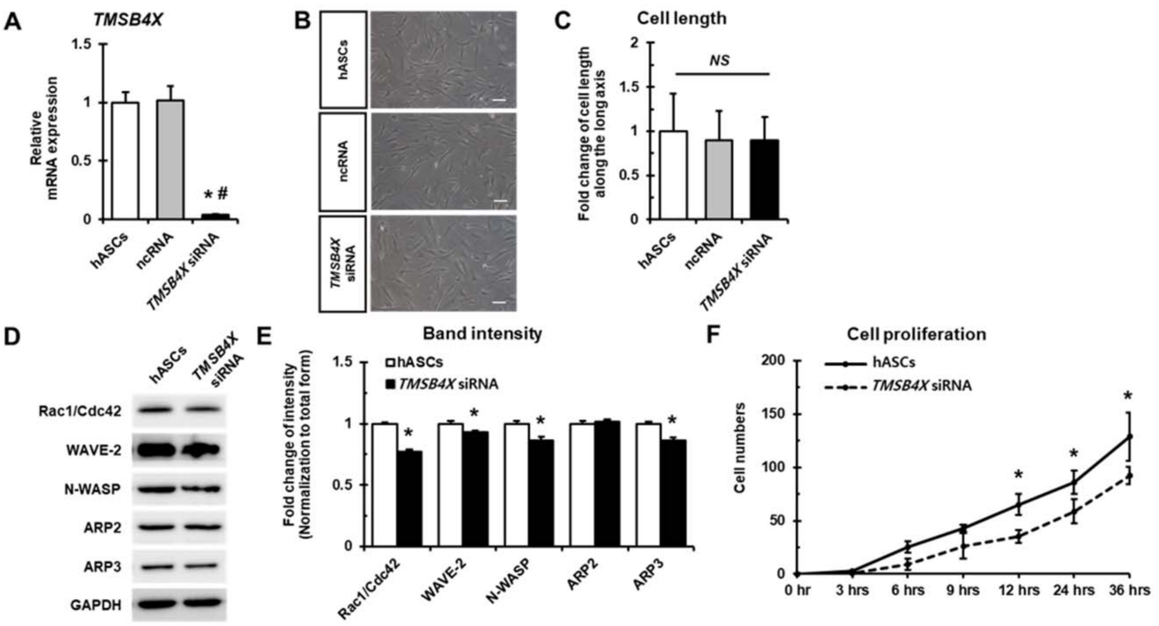

Figure S1. Effects of *TMSB4X* knockdown in hASCs. (A) Relative mRNA expression of *TMSB4X* after 24 hrs of transfection with ncRNA or *TMSB4X* siRNA (100 nM), as evaluated by real-time PCR. *$p < 0.05$ *vs.* hASCs; $^{\#}p < 0.05$ *vs.* ncRNA. (B) Representative phase-contrast images and (C) quantitative analysis of cell length along the long axis after 24 hrs of transfection with ncRNA or *TMSB4X* siRNA. Scale bar = 100 μm; *NS*, not significant. (D, E) Western blot analysis of proteins associated with actin polymerization, such as Ras-related C3 botulinum toxin substrate 1 (Rac1)/cell division cycle 42 (Cdc42), WASP family protein member 2 (WAVE-2), neural WASP (N-WASP), actin-related protein 2 (ARP2), and ARP3 in hASCs and *TMSB4X* siRNA-transfected hASCs. Band intensities were normalized to that of GAPDH. (F) Cell proliferation of hASCs and *TMSB4X* siRNA-transfected hASCs were evaluated by cell count at 3, 6, 9, 12, 24, and 36 hours after seeding. *$p < 0.05$ *vs.* hASCs.

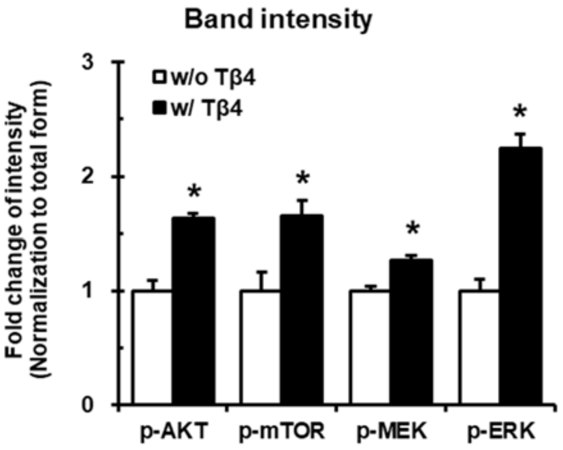

Figure S2. Quantification of western blot results. Band intensity of each phosphorylated protein was normalized to that of the respective total protein (Figure 2E). *$p < 0.05$ *vs.* w/o Tβ4. w/ Tβ4, with Tβ4 treatment; w/o Tβ4, without Tβ4 treatment.
